# Marigold, *Tagetes patula*, a Trap Plant for Western Flower Thrips, *Frankliniella occidentalis,* in Ornamental Bedding Plants Under Controlled Greenhouse Conditions

**DOI:** 10.3390/insects16030319

**Published:** 2025-03-19

**Authors:** Cheryl Frank Sullivan, Bruce L. Parker, Margaret Skinner

**Affiliations:** Entomology Research Laboratory, Department of Agriculture, Landscape and Environment, College of Agriculture and Life Sciences, University of Vermont, 661 Spear Street, Burlington, VT 05405, USA; bparker@uvm.edu (B.L.P.); mskinner@uvm.edu (M.S.)

**Keywords:** western flower thrips, marigold, trap plant, greenhouse ornamentals, integrated pest management, cultural control

## Abstract

Western flower thrips (WFT), *Frankliniella occidentalis* (Pergande) [Thysanoptera: Thripidae], is a destructive pest of greenhouse ornamentals. Flowering yellow marigolds, *Tagetes patula* (L.) [Asterales: Asteraceae], have been shown to be attractive to WFT, implicating their suitability as a trap plant. However, functionality may vary in part due to crop variety, the growth stage of the crop in which the marigold trap plant is deployed and whether or not the crop plants have flowers present. The attractiveness of yellow marigolds was tested within several varieties of mature, flowering ornamental bedding plants that were flowering or had their flowers removed: *Calibrachoa* spp. *Petunia* spp. *Verbena* spp., *Osteospermum* spp., *Impatiens haekeri* and other marigolds. Trials were conducted in cages under controlled greenhouse conditions for six weeks. The effectiveness of orange vs. yellow marigolds as a trap plant was also assessed. The results showed that WFT were attracted to flowering marigolds and, over time, were present in greater numbers on these than crop plants for all crop species and varieties tested at varying levels of significance. Yellow marigold trap plants were the least effective when deployed within other marigold varieties. In crops with flowers, it took up to five weeks for the number of WFT on trap plants to surpass numbers on crop plants. In contrast, in crop varieties with their flowers removed, trap plants attracted WFT earlier, within one to two weeks, and harbored them in greater numbers over time. Orange, flowering marigolds were a less effective trap plant compared to yellow marigolds. These results confirm that flowering yellow marigolds are attractive to WFT and have potential as a trap plant in greenhouse ornamentals, particularly when crop plants do not have flowers.

## Simple Summary

Western flower thrips (WFT) are destructive pests of greenhouse ornamentals. Flowering yellow marigolds are attractive to WFT and have value as trap plants for greenhouse IPM. Marigold trap plant functionality can vary in part due to crop variety, the growth stage of the crop in which it is deployed and the presence of flowers. If used under commercial greenhouse conditions, trap plants would likely be most effective early in the crop production cycle when crop plants are in a vegetative state and when flowers are not present. Regular scouting of trap plants is recommended to prevent the system from becoming a source of pests to the crop.

## 1. Introduction

*Frankliniella occidentalis* (Pergande) [Thysanoptera: Thripidae], western flower thrips (WFT), is one of the most challenging pests to manage on crops grown under protected culture [1]. Their small size, high reproductive potential and ability to rapidly develop pesticide resistance lead to substantial economic losses [2]. Due to resistance from the overuse of chemical pesticides, the development of integrated pest management (IPM) programs that rely on a combination of strategies for WFT suppression is essential [3,4,5,6].

Cultural controls are defined as “changes in crop production methods that affect pests and/or diseases directly, or indirectly through an effect on their natural enemies/antagonists and/or an effect on (induced) crop resistance” [7]. The use of cultural controls as part of IPM may reduce thrip populations on crop plants [6,8]. Plant-mediated IPM systems (i.e., indicator, trap, banker, habitat or guardian plants) are cultural control strategies that utilize plants in combination with other IPM practices to manage pests [9]. Trap crops are used specifically to attract pests from the crop [10,11,12]. Once attracted, the pests can be managed by trap plant disposal or targeted suppression with natural enemy releases or insecticide applications [13,14,15].

Trap plants (real and artificial) using plants in the Asteraceae (daisy) family have shown success in manipulating the behavior of thrips away from crops in field and greenhouse production [13,16,17,18,19]. Examples include flowering yellow chrysanthemums, *Dendranthema grandiflora* (Tzvelev) [14,16], and marigolds, *Tagetes patula* (L.) [13], to attract WFT from greenhouse-grown bedding plants. Although marigolds have been shown to be attractive to WFT in commercial greenhouses [13], it is unclear how the specific crop variety and the presence of flowers affect marigold attractiveness to WFT. Therefore, the goal of this study was to assess the attractiveness of flowering yellow marigolds placed within different ornamental plant varieties in their mature stage with flowers and with flowers removed under controlled greenhouse conditions.

## 2. Materials and Methods

### 2.1. Experimental Design and Data Collection

Six ornamental bedding plant species (two varieties each) were tested: *Calibrachoa* spp. [Solanales: Solanaceae]; *Petunia* spp. [Solanales: Solanaceae]; Vervain, *Verbena* spp. [Lamiales: Verbenaceae]; African daisies, *Osteospermum* spp. [Asterales: Asteraceae]; New Guinea impatiens, *Impatiens haekeri* [Ericales: Balsaminaceae]; and marigold, *Tagetes patula* [Asterales: Asteraceae] (Table 1).

The rooted ornamental plant cuttings were in 72-cell liners when received (Pleasant View Gardens, Inc., Loudon, NH, USA). The plants were transplanted into Metro-Mix^®^360 (Sun Gro Horticulture, Agawam, MA, USA) within 11.43 cm square plastic pots (Landmark Plastics, Akron, OH, USA). Marigolds (variety ‘Hero Yellow’) were grown from seed (Ball Horticultural Company, West Chicago, IL, USA) in 17.78 cm dia. plastic azalea pots (Dillen, The HC Companies, Twinsburg, OH, USA). Plants were grown and the experiments conducted within a temperature-controlled glass greenhouse (5.4 × 6.1 m) at ~23–25 °C, in Burlington, VT, USA under natural light conditions.

**Figure 1:**
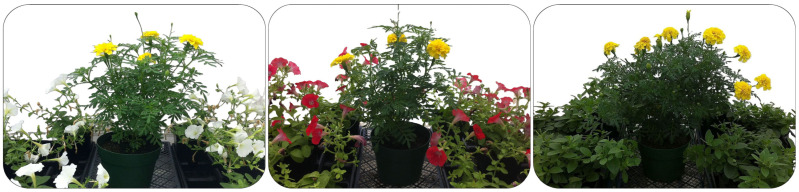
Examples of flowering yellow marigold trap plants in white (**left**) and red (**center**) *Petunias* with flowers and within one of the treatments with flowers removed (**right**) held within cages.

Trials were conducted for each ornamental crop species separately over four years (2013–2016) between January and September (two species tested per season). Twelve insect cages (0.75 × 0.75 × 0.6 m), made from thrip-proof screening, were used for the experiment. Each cage contained six mature flowering crop plants and was randomly assigned a treatment (flowers or flowers removed) for each variety. Each treatment (four total) was replicated three times within the greenhouse. The plants were grown to flowering, after which the plants for the non-flowering treatment had their blossoms removed prior to the start of the experiment. All plants were inspected visually for pests by gently tapping the entire plant canopy over a piece of laminated white paper (28 × 22 cm) prior to artificially infesting them with WFT. Each plant received two 24 h old laboratory-reared mated females (12 WFT/cage). After one week, a flowering marigold trap plant (~20.32–25.4 cm initial height) was placed in the center of the cage (Figure 1).

The number of WFT adults and larvae for each plant within the cages were counted weekly for six weeks by gently tapping the plants over the laminated white paper. After sampling, dislodged WFT were returned to the foliage of their respective plants. Any blossoms on the crop plants in the without-flower treatments were removed and placed in a tray at the base of the plant, which ensured minimal loss of WFT. This maintained plants without flowers throughout the experiment. Marigold foliage height above the crop canopy was maintained at ~20.32–25.4 cm by placing the marigold on an overturned 17.78 cm dia. plastic azalea pot in treatments with taller crop plants. Foliar damage from thrips feeding (silvering or stippling on the leaf surface, leaf curling, stunting and distortion) on each plant was assessed visually using the following rating scale: 0: no damage; 1: <10% foliage damaged; 2: 10–25% foliage damaged; 3: 26–50% foliage damaged; 4: 51–75% foliage damaged; and 5: >76% foliage damaged. The same researcher conducted the inspections for consistency in data collection.

In the final year, we examined the effectiveness of the flowering, orange marigold variety as a trap plant compared to the yellow variety placed within yellow marigolds with and without flowers (the same varieties as described above). Plants were grown and infested with WFT, and the data were collected in the same manner as described above, except the experiment duration was four weeks. This was because plants in a few treatments succumbed to infestations of spider mites, *Tetranychus urticae* Koch (Arachnida: Acari: Tetranychidae), prompting early termination of the experiment.

### 2.2. Data Analysis

The number of WFT dislodged from the plants and the damage ratings were analyzed separately for each crop species. Within each cage, the number of WFT on the six crop plants were averaged for each sampling date to obtain the mean number of WFT per crop plant to compare with the total number of WFT observed on the single marigold trap plant. The number of total WFT (adults + larvae) were analyzed over time separately using a general linear model with repeated measures. The between-subject factor was the treatments, which were the crop color and flowering stage flower status (with flowers or with flowers removed) combinations for the different plant species. For the orange vs. yellow trap plant trial, trap plant color was used in place of crop color. The within-subject factors were plant type (crop or marigold trap) within each observation time (1–6 weeks). If Mauchly’s test of sphericity was violated, Greenhouse–Geisser or Huynh–Feldt corrections were applied to correct the degrees of freedom of the F-distribution depending on if the estimated epsilon (ε) value was below or above 0.75, respectively. Pairwise comparisons between the marigold trap plant and the crop variety within each week and for overall treatment differences were assessed using the Bonferroni adjustment for multiple comparisons. The presented data are untransformed and reported in their original form. A significance level of (α = 0.05) was used for all comparisons. Analyses were conducted using IBM-SPSS v.29 (IBM, Armonk, NY, USA) and figures produced with Prism v.10 (GraphPad Software, Boston, MA, USA).

## 3. Results

The results showed that when flowering yellow marigolds were used in different species and varieties of ornamental crops with and without flowers as a trap plant, the number of WFT eventually exceeded the number on crop plants at varying levels of significance over time except for when they were used in other marigold varieties. In general, trap plants attracted WFT earlier than crop plants. The differences in WFT numbers between the trap and crop plants were more significant over time in crops that had their flowers removed.

### 3.1. Calibrachoa

**Figure 2:**
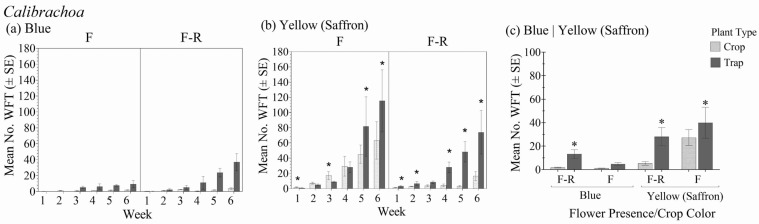
Mean number of WFT (adults + larvae) ± SE on flowering yellow marigold trap plants in the flowering stages of blue (**a**) and yellow/saffron (**b**) *Calibrachoa* with flowers (F) and with flowers removed (F-R) each week for 6 weeks and averaged over the 6-week experiment duration (**c**). An * indicates a significant difference between the trap and crop plant within crop flowering status and color treatment groups (Bonferroni, *p* < 0.05).

Within the different *Calibrachoa* crop color variety and flower status combinations, there was a significant interaction between plant type (crop vs. trap plant) over the six-week period in relation to the total number of WFT (adults + larvae) (F_1.9,14.9_ = 9.12; *p* = 0.003) (Figure 2a,b). In general, there was no significant interaction between the crop color–flowering status and plant type (crop vs. trap plant) (F_3,8_ = 2.89; *p* = 1.02), but the effect of plant type within the crop color–flowering status treatments was significant (F_1,8_ = 29.8; *p* < 0.001) (Figure 2c). There were more WFT on the trap plants than on crop plants within each color–flowering status combination except in the blue flowering variety. When flowers were present, it took five weeks in the yellow and three weeks in the blue for WFT numbers on trap plants to exceed those on crop plants. In contrast, when flowers were absent, more WFT were found on the trap plants after one week. The differences in WFT numbers between the crop and trap plants were greatest for yellow *Calibrachoa*.

In the cages with yellow-flowering *Calibrachoa,* there were significantly more WFT on crop plants within the first three weeks, whereas there were significantly more WFT on trap plants at six weeks. In contrast, when flowers were absent, there were significantly more WFT adults on the trap plants early on, resulting in significantly greater total numbers at six weeks. At six weeks, trap plants in crops with no flowers harbored an average of 145% more WFT than the crop, whereas trap plants in crops with flowers had 98% more WFT than the crop.

When foliar damage was assessed, there were significant differences over time between the *Calibrachoa* crop plants and trap plants (F_5,40_ = 24.42; *p* < 0.001). At six weeks, the trap plant had significantly greater foliar damage than the crop plants in both blue and yellow *Calibrachoa* without flowers. However, no significant differences in damage were observed between the *Calibrachoa* with flowers and trap plants (Table 2(a)).

### 3.2. Impatiens

**Figure 3:**
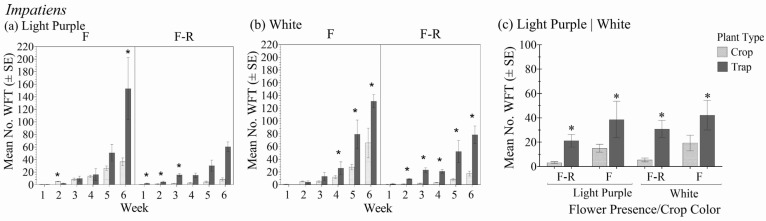
Mean number of WFT (adults + larvae) ± SE on flowering yellow marigold trap plants in the flowering stages of purple (**a**) and white (**b**) *Impatiens* with flowers (F) and with flowers removed (F-R) each week for 6 weeks and averaged over the 6-week experiment duration (**c**). An * indicates a significant difference between the trap and crop plant within crop flowering status and color treatment groups (Bonferroni, *p* < 0.05).

There was a significant interaction between plant type (crop vs. trap plant) over the six-week period within the different crop color variety and flower status combinations in relation to the total number of WFT (F_1.8,14.0_ = 24.37; *p* < 0.001) (Figure 3a,b). In general, there was no significant interaction between the *Impatiens* crop color–flower status and plant type (crop vs. trap plant) (F_3,8_ = 0.26; *p* = 0.852), but the effect of plant type was significant (F_1,8_ = 53.5; *p* < 0.001) (Figure 3c).

When flowers were present, it took three weeks for the total WFT numbers on trap plants to exceed those on crop plants. In contrast, when flowers were absent, WFT were found on the trap plants after one week. The differences in total WFT between the crop and trap plants became more significant over time for white *Impatiens* both with and without flowers. However, in the purple variety, the trap plant was significantly more attractive early on and only when flowers were not present. At six weeks, trap plants in the *Impatiens* without flowers harbored an average of 140% more WFT than the crop, whereas marigolds in the crops with flowers had an average of 95% more WFT than the crop.

When foliar damage was assessed, there were significant differences over time between the *Impatiens* and associated trap plants (F_1.5,12.4_ = 22.86; *p* < 0.001). After six weeks, the trap plant had greater foliar damage than the crop plants in all crop color and flower status combinations, and the differences were significant in both *Impatiens* varieties without flowers (Table 2(b)).

### 3.3. Osteospermum

**Figure 4:**
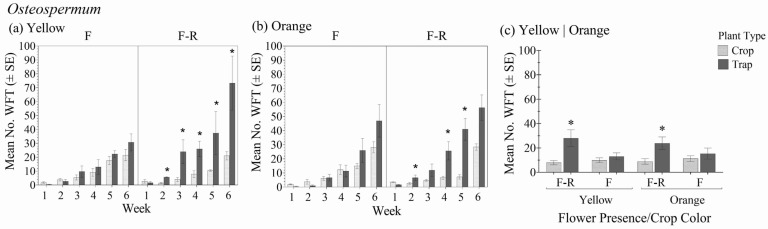
Mean number of WFT (adults + larvae) ± SE on flowering yellow marigold trap plants in the flowering stages of yellow (**a**) and orange (**b**) *Osteospermum* with flowers (F) and with flowers removed (F-R) each week for 6 weeks and averaged over the 6-week experiment duration (**c**). An * indicates a significant difference between the trap plant and the crop plant within crop flowering status and color treatment groups (Bonferroni, *p* < 0.05).

Within the different crop color variety and flower status combinations, there was a significant interaction between plant type (crop vs. trap plant) over the six-week period in relation to the total number of WFT (F_1.8,14.4_ = 13.03; *p* < 0.001) (Figure 4a,b). In general, there was no significant interaction between the *Osteospermum* crop color–flower status and plant type (crop vs. trap plant) (F_3,8_ = 2.73; *p* = 0.114), but the effect of plant type was significant (F_1,8_ = 17.89; *p* = 0.003) (Figure 4c).

Over time, more WFT were observed on trap plants than on *Osteospermum* crop plants within each color–flower status combination. In the flower treatments, it took three weeks for total WFT numbers on trap plants to exceed those of the crop plants. In contrast, when crop plants had no flowers, WFT were found on trap plants after two weeks. Although the marigolds eventually harbored more WFT than the crops with flowers, differences were not significant. In contrast, the differences in total WFT between the crop varieties with flowers and the trap plants were significant within two weeks and remained significant until week six in the yellow *Osteospermum* variety. At six weeks, trap plants in the crops without flowers harbored an average of 89% more WFT than the crop, whereas trap plants in the flowering crops had up to 43% more WFT than the crop.

There were significant differences over time in foliar damage between the *Osteospermum* crop plants and associated trap plants (F_5,40_ = 12.52; *p* < 0.001). At six weeks, the marigold had greater foliar damage than the crop plants in all crop color and flower status combinations, but the difference was only significant in the yellow variety without flowers (Table 2(c)).

### 3.4. Petunia

**Figure 5:**
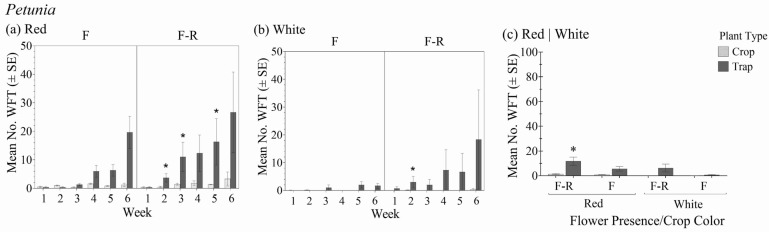
Mean number of WFT (adults + larvae) ± SE on flowering yellow marigold trap plants in the flowering stages of red (**a**) and white (**b**) *Petunia* with flowers (F) and with flowers removed (F-R) each week for 6 weeks and averaged over the 6-week experiment duration (**c**). An * indicates a significant difference between the trap and crop plant within crop flowering status and color treatment groups (Bonferroni, *p* < 0.05).

Of all the test crops, *Petunia* had the lowest WFT population establishment. Within the different crop color variety and flower status combinations, there was a significant interaction between plant type (crop vs. trap plant) over the six-week period in relation to the total number of WFT (F_1.2,9.3_ = 7.04; *p* = 0.023) (Figure 5a,b). In general, there was no significant interaction between the *Petunia* crop color–flower status and plant type (crop vs. trap plant) (F_3,8_ = 0.991; *p* = 0.444), but the effect of plant type was significant (F_1,8_ = 7.8; *p* = 0.023) (Figure 5c).

Over time, greater numbers of WFT were observed on trap plants than the *Petunia* crop plants within each color–flower status combination. WFT populations on the red *Petunia* variety was greater than the white. When crops had flowers, it took three weeks for total WFT numbers on trap plants to exceed those of the crop plants. However, no significant differences within week between the trap and crop plants were observed. In contrast, when crops did not have flowers present, WFT were observed on the trap plants after one week in the white variety and after two weeks in the red and were significantly greater than those on the crop plants for both colors. At six weeks, trap plants in the crops with no flowers harbored an average of 174% more WFT than the crop whereas trap plants in crops with flowers had up to 89% more WFT than the crop.

When foliar damage was assessed, there were significant differences over time between the *Petunia* crop plants and associated trap plants (F_5,40_ = 10.71; *p* < 0.001). At six weeks, the trap plants had greater foliar damage than the crop plants in all crop color and flower status combinations, but the difference was only significant in the white variety without flowers (Table 2(d)).

### 3.5. Verbena

**Figure 6:**
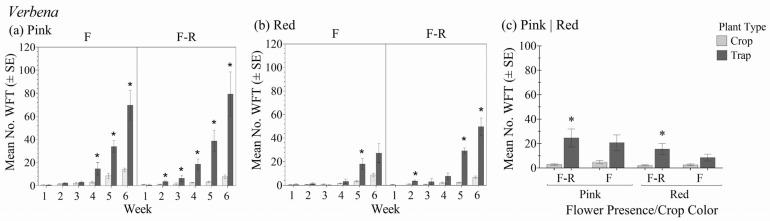
Mean number of WFT (adults + larvae) ± SE on flowering yellow marigold trap plants in the flowering stages of pink (**a**) and red (**b**) *Verbena* with flowers (F) and with flowers removed (F-R) each week for 6 weeks and averaged over the 6-week experiment duration (**c**). An * indicates a significant difference between the trap and crop plant within crop flowering status and color treatment groups (Bonferroni, *p* < 0.05).

There was a significant interaction between plant type (crop vs. trap plant) over the six-week period within the different crop color variety and flower status combinations in relation to the total number of WFT (F_1.6,12.4_ = 49.76; *p* < 0.001) (Figure 6a,b). In general, there was a significant interaction between the *Verbena* crop color-flower status and plant type (crop vs. trap plant) (F_3,8_ = 4.82; *p* = 0.033), and the effect of plant type was also significant (F_1,8_ = 90.54; *p* < 0.001) (Figure 6c).

Over time, greater numbers of WFT were observed on the trap plants than on *Verbena* crop plants within each color–flower status combination. In all growth stages, it took up to two weeks for total WFT numbers on marigold trap plants to exceed those of the crop plants. Marigold trap plants were especially attractive in the pink variety in both flower status treatments. The trap plant harbored significantly more WFT from two through six weeks when no flowers were present and four through six weeks when flowering. For the red variety, the trap plant harbored greater numbers of WFT on more weeks, especially after five and six weeks. At six weeks, marigold trap plants in crops without flowers harbored an average of 159% more WFT than the crop, whereas marigolds in the crops with flowers had up to 119% more WFT than the crop.

There were significant differences in foliar damage over time between the *Verbena* crop plants and associated trap plants (F_5,40_ = 22.14; *p* < 0.001). At 6 weeks, the marigold trap plants had greater foliar damage than the crop plants in all crop color and flower status combinations and the differences were significant for both varieties with no flowers (Table 2(e)).

### 3.6. Tagetes

**Figure 7:**
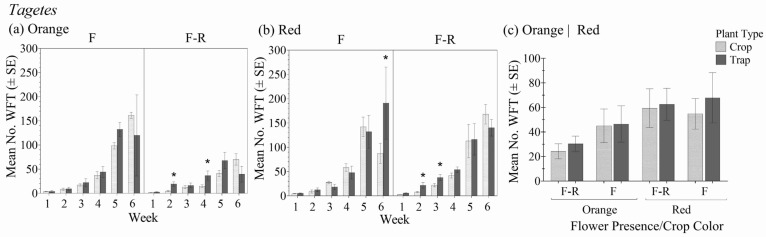
Mean number of WFT (adults + larvae) ± SE on flowering yellow marigold trap plants in the flowering stages of orange (**a**) and red (**b**) *Tagetes* with flowers (F) and with flowers removed (F-R) each week for 6 weeks and averaged over the 6-week experiment duration (**c**). An * indicates a significant difference between the trap and crop plant within crop flowering status and color treatment groups (Bonferroni, *p* < 0.05).

Within the different crop color variety and flower status combinations, there was no significant interaction between plant type (crop vs. trap plant) over the six-week period in relation to the total number of WFT (F_4.1,28.8_ = 0.154; *p* = 0.962) (Figure 7a,b). In general, there was no significant interaction between the *Tagetes* crop color–flower status and plant type (crop vs. trap plant) (F_3,7_ = 0.27; *p* = 0.842). However, unlike the other crop varieties, the effect of plant type was not significant (F_1,7_ = 1.81; *p* = 0.221) (Figure 7c).

The number of WFT observed on the trap plants was not significantly different from those on the crop plants within each color–flower status combination, with a few notable exceptions within specific weeks. In the crops without flowers, there were significantly more WFT on the trap plants earlier during the six-week period (weeks two and four for the orange variety and weeks two and three for the red). However, by the end of the six-week period, WFT numbers were higher on the crop plants than the trap plants. This was also observed in the orange variety with flowers. In contrast, by week six in the red variety with flowers, there were significantly more WFT on the yellow trap plant than the crop, whereas WFT were higher on the crop rather than the trap plant until that point. Unlike the other crop plant varieties, after six weeks, yellow trap plants in red and orange *Tagetes* without flowers harbored an average of 37% fewer WFT than the crop. However, trap plants in the orange crop with flowers had 29% fewer WFT than the crop, whereas in the red variety with flowers, the trap plant had 52% more WFT than the crop.

When foliar damage was assessed, there were significant differences over time between the crop plants and associated trap plants (F_4.4,13.3_ = 3.62; *p* = 0.013). At six weeks, the trap plants had equal or greater foliar damage than the crop plants in all crop color and flower status combinations, and the differences were significant in the orange variety with and without flowers (Table 2(f)). At six weeks, the numbers of WFT caused significant damage in the orange varieties.

### 3.7. Trap Plant Color

**Figure 8:**
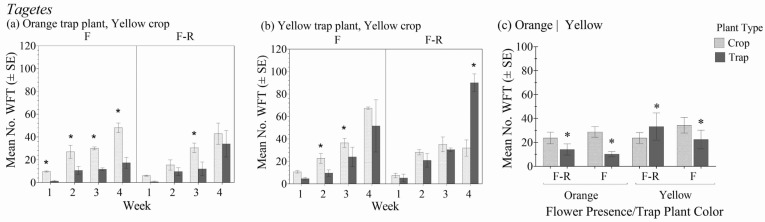
Mean number of WFT (adults + larvae) ± SE on flowering orange (**a**) or yellow (**b**) marigold trap plants in the flowering stage of yellow *Tagetes* crop plants with flowers (F) and with flowers removed (F-R) each week for 4 weeks and averaged over the 4-week experiment duration (**c**). An * indicates a significant difference between the trap and crop plant within crop flowering status and color treatment groups (Bonferroni, *p* < 0.05).

Within the different trap crop color variety and flower status combinations, there was no significant interaction between plant type (crop vs. trap plant) over the four-week period in relation to the number of WFT (F_3.4,7.83_ = 2.37; *p* = 0.163) (Figure 8a,b). In general, there was a significant interaction effect between the trap crop color treatments and plant type (crop vs. trap plant), (F_3,7_ = 14.9; *p* = 0.002), and the effect of plant type was significant (F_1,7_ = 23.1; *p* = 0.002) (Figure 8c).

The number of WFT was consistently significantly greater on flowering yellow crop plants than the orange and yellow trap plants. There was also limited attractiveness of the flowering trap plants used in crops without flowers, with one exception when the yellow trap plant was used within yellow crop plants without flowers after four weeks. This was the only instance when the trap plant was more attractive than the crop plant within a given time point. These results are similar to the other trials in *Tagetes* where WFT were generally not significantly attracted to the trap plants. This suggests that marigolds of other colors (such as orange) may have limited effectiveness as a trap plant and that marigold color aids in WFT attraction.

## 4. Discussion

Earlier studies suggested yellow marigolds are attractive to WFT when deployed in commercially grown bedding plants as either a trap or guardian plant [13]. However, it is unclear how specific crop varieties and the presence of flowers affect marigold attractiveness to WFT. The results reported herein confirm the attractiveness of yellow marigolds, reaffirming their value as a trap plant for use in greenhouse ornamentals. The attractiveness of flowering marigold trap plants is likely the greatest early in the season when fewer crop plants are in bloom, as was observed in other studies of flowering chrysanthemums [16]. These results showed that WFT (adults primarily) were generally observed earlier on marigold trap plants within crop plants without flowers compared to crop plants that had flowers. In addition, WFT stayed on the trap plants over time in numbers greater than the crop plants. The exception was when marigolds were used as a trap crop in other marigold varieties.

Why marigolds are attractive to WFT is complex. Visual and olfactory cues are likely involved. Volatiles emitted from flowers and foliage, pollen and nectar availability and plant life stage, quality and color are important factors influencing WFT plant selection. These vary among plant species and color shades based on their spectral properties [20,21,22,23,24]. Western flower thrips are generally attracted to yellow and blue, colors commonly used for sticky monitoring card tools within greenhouse IPM [6,23,25]. The flowers of yellow varieties of plants in the Asteracea family such as transvaal daisy (*Gerbera* sp.) and chrysanthemum (*Dendranthema* sp.) have been reported to be preferred over other colors when tested within the different genera [20]. Yellow is also the color that has been tested in plant-mediated IPM systems using chrysanthemums and marigolds in greenhouse bedding plants [13,14,16]. The attraction of WFT to marigolds indicates they are a host plant for reproduction. A study by Cao et al. [21] examined the suitability and preference of different plant hosts to WFT. *Tagetes erecta* (African marigold) was among the most suitable flowering plant hosts, preceded by *Rosa* spp. In a related study, it was determined that WFT were attracted to *T. erecta* volatiles and varied with different visual color cues and sex [22]. Our findings build on these reports and further confirm that WFT attraction to flowering marigolds is strongly color-related and that marigolds are a suitable host for WFT.

Cultural controls must be implemented early in the cropping season to prevent pest outbreaks [12]. For greenhouse-grown bedding plants, strategies that target non-flowering crops would be ideal prior to WFT population buildup. Floral resources are important for WFT, providing pollen and nectars to enhance fecundity and protected hiding and mating locations [1,26,27]. Thrips’ attraction to plants depends on several factors and preferences depend on whether the plant is for reproduction or food [27]. Plant species and cultivar, growth stage, and flower size, shape, stage, and color all may influence attractiveness [24,28,29]. Attraction to flowering instead of non-flowering plant stages, which resulted in increased reproduction, has been reported in several studies. For example, it has been demonstrated that adult WFT prefer flowering over non-flowering chrysanthemums, which led to greater reproductive success [28]. Plant height was also important, with taller cultivars showing more damage. In a different study, it was observed that WFT pupated on-plant when chrysanthemums were flowering, in contrast to non-flowering plants, where a greater proportion chose to pupate in the soil [26]. Ren et al. [24] reported that several flowering plants emitted more volatiles and attracted more WFT than in their non-flowering stages.

IPM programs use a combination of practices to reduce pests and grower reliance on synthetic chemical pesticides [3,12]. Scouting is an integral part of IPM that allows for early intervention [5]. Because WFT can build up rapidly on marigold trap plants and marigolds are also highly attractive to spider mites [3,30], routine monitoring by growers or their scouts would be necessary to prevent the trap plants from serving as a reservoir for pests that could move to the crop. Pests on trap plants can be managed using a variety of tactics like plant removal and disposal, releases of natural enemies or treatment with an insecticide [13,14].

In summary, it was determined that yellow flowering marigolds attract WFT off crop plants, and the extent of attractiveness depended on crop variety and whether the crop had flowers. Marigolds were generally more attractive when placed in crops without flowers, and marigolds were attractive regardless of flower presence or color. Because it is unknown how the interaction between visual and olfactory cues affects WFT behavior in the presence of marigold trap plants, further investigations are encouraged. More in-depth studies of trap plant longevity before acting as a source of WFT to the crop is also recommended. In addition, their effectiveness under commercial greenhouse settings should continue to be investigated. This would improve the use of marigolds as a WFT trap plant in greenhouse-grown bedding plants.

Implementing IPM programs for WFT and other greenhouse ornamental pests is challenging and evolves as new strategies and pest challenges arise [3,4,5,12]. The incorporation of plant-mediated IPM systems, such as trap plants, to aid in the detection and suppression of WFT is a valuable and cost-effective IPM tool for reducing reliance on chemical pesticides and aiding in resistance management. Further investigations of these systems under protected culture should be encouraged under real-world conditions. The refinement of promising trap plant candidates, such as yellow marigolds, or even other marigold colors, to demonstrate effectiveness in terms of cost and control would increase grower adoption and successful implementation in greenhouse ornamentals.

## Figures and Tables

**Table 1:** Ornamental plants tested against Hero^®^ yellow marigold, *Tagetes patula*, trap plants.

Genus and Species	Series	Color
*Calibrachoa*, hybrid	Superbells^®^	Saffron (Yellow)
*Calibrachoa*, hybrid	Superbells^®^	Blue
*Impatiens hawkeri*	Infinity^®^	Light Purple
*Impatiens hawkeri*	Infinity^®^	White
*Osteospermum*, hybrid	Symphony^®^	Yellow
*Osteospermum*, hybrid	Symphony^®^	Orange
*Petunia*, hybrid	Supertunia^®^	White
*Petunia*, hybrid	Supertunia^®^	Red
*Verbena*, hybrid	Superbena^®^	Royale Iced Cherry (Red)
*Verbena*, hybrid	Superbena^®^	Royale Peachy Keen (Pink)
*Tagetes patula*	Durango^®^	Red
*Tagetes patula*	Little Hero^®^	Orange

**Table 2:** Mean foliar damage ratings between different color varieties of ornamental crop plants in their flowering stages with flowers (F) and with flowers removed (F-R) and their associated marigold trap plants at six weeks.

Treatment (Crop Plant Color and Stage)	Mean Foliar Damage Rating ^1^		
	Crop Plant	Marigold Trap Plant	*p*-Value
(a) *Calibrachoa*			
F Yellow	4.00 ± 1.00	4.00 ± 1.00	1.000
F-R Yellow	2.33 ± 0.67	4.00 ± 0.58 *	0.002
F Blue	1.00 ± 0.00	1.00 ± 0.00	1.000
F-R Blue	1.00 ± 0.00	2.33 ± 0.67 *	0.007
(b) *Impatiens*			
F Purple	1.83 ± 0.17	4.00 ± 1.00	0.072
F-R Purple	0.78 ± 0.62	3.33 ± 0.88 *	0.040
F White	2.72 ± 0.15	4.00 ± 1.00	0.256
F-R White	1.33 ± 0.33	4.67 ± 0.33 *	0.013
(c) *Osteospermum*			
F Yellow	1.67 ± 0.33	2.67 ± 0.33	0.147
F-R Yellow	2.33 ± 0.33	4.00 ± 0.58 *	0.028
F Orange	1.67 ± 0.33	2.67 ± 0.67	0.147
F-R Orange	2.67 ± 0.33	4.00 ± 0.58	0.065
(d) *Petunia*			
F White	0.00 ± 0.00	0.67 ± 0.33	0.213
F-R White	0.17 ± 0.17	1.33 ± 0.88 *	0.045
F Red	1.00 ± 0.00	2.00 ± 0.00	0.077
F-R Red	1.33 ± 0.33	2.33 ± 0.67	0.077
(e) *Verbena*			
F Red	1.67 ± 0.33	2.33 ± 0.33	0.169
F-R Red	1.00 ± 0.00	3.67 ± 0.33 *	<0.001
F Pink	3.00 ± 0.00	3.67 ± 0.33	0.169
F-R Pink	2.33 ± 0.33	4.00 ± 0.58 *	0.005
(f) *Tagetes*			
F Red	5.00 ± 0.00	5.00 ± 0.00	1.000
F-R Red	5.00 ± 0.00	5.00 ± 0.00	1.000
F Orange	4.00 ± 0.00	5.00 ± 0.00 *	0.002
F-R Orange	3.72 ± 0.36	4.33 ± 0.67 *	0.008

^1^ Foliage damage rating scale: 0: no damage; 1: <10%; 2: 10–25%; 3: 26–50%; 4: 51–75%; and 5: >76%. Means within rows with an * are significantly different (Bonferroni, *p* < 0.05).

## Data Availability

The raw data supporting the conclusions of this article will be made available by the authors on request.
